# How to Evaluate Kidney Function in Elite Endurance Athletes: Pros and Cons of Different Creatinine-Based Formulas

**DOI:** 10.3390/jcm14092955

**Published:** 2025-04-24

**Authors:** Giuseppe Di Gioia, Armando Ferrera, Andrea Serdoz, Alessandro Spinelli, Roberto Fiore, Lorenzo Buzzelli, Domenico Zampaglione, Maria Rosaria Squeo

**Affiliations:** 1 Institute of Sports Medicine and Science, National Italian Olympic Committee, Largo Piero Gabrielli, 1, 00197 Rome, Italy; armando.ferrera95@gmail.com (A.F.); andreaserdoz@gmail.com (A.S.); alessandro.spinelli1@gmail.com (A.S.); roberto.fiore@mail.com (R.F.); domzamp1@gmail.com (D.Z.); mariarosaria.squeo@coni.it (M.R.S.); 2 Department of Movement, Human and Health Sciences, University of Rome “Foro Italico”, Piazza Lauro De Bosis, 15, 00135 Rome, Italy; 3 Department of Cardiovascular Sciences, Fondazione Policlinico Campus Bio Medico di Roma, Via Alvaro del Portillo, 200, 00128 Rome, Italy; lorenzo.buzzelli@unicampus.it

**Keywords:** kidney, athletes, endurance, creatinine, sports medicine

## Abstract

**Background:** Various creatinine-based equations are used to estimate the glomerular filtration rate (eGFR) in athletes, but each has limitations. The aim of our study was to identify the most suitable formula for use in athletes. **Methods:** We evaluated 490 Olympic athletes (27 ± 5.3 yo) with normal values of serum creatinine and no history of kidney diseases. Athletes were divided into those practicing skills and endurance disciplines. The EGFR was calculated with Cockcroft–Gault (CG), MDRD, MCQE and CKD-EPI, and classified as stages G1–G5 according to the Kidney Disease Improving Global Outcomes (KDIGO) GFR categories. **Results:** Endurance athletes showed higher serum creatinine (0.91 ± 0.14 mg/dL vs. 0.88 ± 0.13 mg/dL in skills, *p* = 0.014). The eGFR calculated with the CKD-EPI and MCQE formulas showed no differences between the groups. The CG formula produced a lower eGFR for endurance athletes (113.6 ± 27 mL/min/1.73 m^2^) compared to skills athletes (122.6 ± 30.8, *p* = 0.008), while MDRD produced higher values for endurance athletes (129.3 ± 25.8 vs. 122.6 ± 24 mL/min/1.73 m^2^, *p* = 0.004). According to CKD-EPI, all athletes were in G1, while with MCQE, 0.5% of skills athletes and 1% of endurance athletes were in G2. With the CG formula, a significant percentage of athletes were in G2 (13.2% of skills athletes and 18.5% of endurance athletes, *p* = 0.125). With the MDRD formula, 29 athletes (5.9%) were in G2 (6% for skills athletes and 5.8% for endurance athletes, *p* = 0.927). **Conclusions:** CKD-EPI and MCQE showed better stability and reliability, making them the most suitable for kidney function evaluation in athletes.

## 1. Introduction

Quantitative assessment of kidney function is a fundamental element in daily clinical practice, as well as in sports medicine, where it is used to assess athletes’ overall health status. This is of special relevance for endurance athletes, particularly those participating in high-volume, high-intensity ultra-running events, as they are at increased risk of acute kidney injury (AKI) [1,2], which raises concerns about the chronic development of progressive renal scarring [3]. In this regard, however, the prevalence of AKI, which appears to be a rapidly self-resolving phenomenon in the absence of medical intervention [4,5,6], varies widely due to differences and inconsistency among the methods used to assess renal function in this setting, with the risk of underestimating the extent of the problem [1,2,7]. Glomerular filtration rate (GFR) is traditionally considered the key index of kidney function, but its precise measurement by inulin or radioisotope studies is invasive, time-consuming, expensive, associated with radiation exposure and technically difficult [8,9,10]. Therefore, in view of the existence of an inverse, nonlinear relationship between GFR and serum creatinine concentration [11], a skeletal muscle catabolite closely influenced by muscle mass and dietary protein intake, several equations have been developed to estimate GFR by using serum creatinine data combined with readily available indices of muscular mass, such as gender, age and weight [9,10,12,13]. Furthermore, it is important to note that eGFR equations are not suitable for the assessment of AKI in athletes, as they assume a steady state of serum creatinine. In athletes, particularly after endurance exercise, creatinine levels may fluctuate rapidly, and eGFR calculations may significantly misrepresent acute changes in kidney function.

The most common equations used in adults are the Cockcroft–Gault equation (CG) [14] and the simplified equation from the Modification of Diet in Renal Disease Study (MDRD) [15]. These equations differ in various aspects, including the predicted index (with CG, creatinine clearance is predicted, which is an estimate of the GFR value plus the tubular secretion, while with MDRD, true GFR is predicted), prediction units (ml/min for CG, which is uncorrected for BSA, as opposed to mL/min × 1.73 m^2^ for MDRD) and variables involved (serum creatinine, gender, age and body weight for CG; serum creatinine, gender, age and race for MDRD). However, estimation is only an approximate calculation and reasons why the measurements might be incorrect are numerous, thus resulting in many criticisms related to the pros and cons of each formula [16]. CG, in fact, has a tendency to underestimate in older-age or underweight individuals and overestimate in overweight/obese individuals, as it includes body weight as an inappropriate indicator of muscle mass, without taking into account the fat component [9,10,12,16,17]. Moreover, having been designed to calculate an estimate of creatinine clearance, which includes both glomerular filtration and tubular secretion, it tends toward overestimation in people with normal renal function [9,16]. Similarly, MDRD has limitations too: being derived from subjects with moderate-to-severe renal failure, it exhibits lower accuracy in subjects without renal disease, with a tendency to underestimate the GFR, especially in those with a normal or slightly increased range of serum creatinine concentration and in females, irrespective of BMI, and underestimates to a greater extent in young subjects [9,10,12,17,18]. Therefore, in light of the reduced performance of MDRD in the healthy population, the Mayo Clinic Quadratic Equation (MCQE), based on serum creatinine, gender and age, was subsequently devised, with the aim of obtaining a more accurate estimate of true GFR in subjects without renal disease [19]. However, the latter equation, despite performing moderately well in comparison with gold-standard radionuclide GFR measurements, has not subsequently demonstrated any performance advantage in clinical practice over MDRD, even in healthy populations, and has shown a tendency to overestimate in males and individuals with a non-low GFR and obesity [12,20]. More recently, in view of the limited precision of the previous formulas, an additional new equation, the Chronic Kidney Disease Epidemiology Collaboration (CKD-EPI), was developed and derived from a population consisting predominantly of young or middle-aged people, with and without kidney disease, using the same four variables as the MDRD equation [21,22]. The CKD-EPI shows improved precision and gives the best overall accuracy in estimating the GFR throughout its whole range, providing a less biased GFR assessment than the MDRD study equation, particularly in middle-aged and female patients and those with GFR levels >60 mL/min/1.73 m^2^, where it reduces the apparent prevalence of chronic kidney disease (CKD) [23,24,25,26,27,28]. Lastly, owing to exercise-induced metabolic adaptations, the interpretation of several biochemical data requires caution in elite athletes, as specific reference values for laboratory parameters have never been established in this population, where those retrieved from the general population, including serum creatinine, are routinely applied. Although creatinine-based GFR estimates overcome some shortcomings of serum creatinine, only a few studies, with limited numbers of participants, have previously evaluated their use and performance in endurance athletes, showing significant variations in the estimated GFR among athletes and the influence of multiple factors in this particular group of individuals.

Hence, the aim of our study was to compare different formulas for eGFR in a large cohort of elite endurance and skill athletes, in order to determine which equation provides the most appropriate estimate of kidney function in this unique population.

## 2. Materials and Methods

The Institute of Sports Medicine and Science in Rome is an establishment of the Italian National Olympic Committee, with the mission of medically evaluating athletes selected for participation in the Olympic Games, World Championships and Mediterranean Games. In the present analysis, we recruited 490 Caucasian elite athletes evaluated before participating in Rio 2016, PyeongChang 2018 and Tokyo 2020 Olympic Games.

Power calculation was performed based on effect sizes and variability reported in previous studies investigating renal function and serum creatinine in athletic populations.

Athletes underwent complete physical examination, anthropometric analysis and complete blood tests. None of the enrolled athletes were taking chronic pharmacological therapy or creatine supplementation. Twenty-three Afro-Caribbean athletes were excluded; of these, five athletes with high blood pressure taking chronic pharmacological therapy were excluded, and two athletes with type 1 diabetes were excluded. All athletes included in our study presented normal values of serum creatinine and no previous history of kidney disease.

Athletes participated in different sports disciplines, and were classified into two groups, according to European Society of Cardiology (ESC) guidelines [29,30]:

(1) Skills: archery, equestrian, golf, shooting, figure skating, sailing, curling, diving, surfing and equestrian sports.
(2) Endurance: cycling, rowing, canoeing, triathlon, long-distance running, long-distance swimming (over 800 m), cross-country skiing, pentathlon, biathlon, Nordic combined and long-distance skating.

Body composition and fat mass percentage were measured using Bioelectric Impedance Analysis (BIA 101 Quantum, Akern, Pisa, Italy) with a constant sinusoidal current at an intensity of 50 kHz and 400 μA. Body height and weight were obtained for each subject, and the body mass index (BMI) was calculated as weight (kg)\height (m)^2^. Body surface area (BSA) was derived with the Mosteller formula [31]. The CV risk factors evaluated in this study were defined as follows: cigarette smoking was defined as regular smoking of at least one cigarette per day; obesity was defined as BMI > 30.

Blood samples were drawn after fasting with an aseptic technique from a vein in the cubital fossa, and were transported to an adjacent laboratory, where analysis was performed on the same day. The following biochemical parameters were assessed: full blood count, ferritin, transferrin, iron, potassium, calcium, magnesium, urate, creatinine, aspartate amino-transaminase (AST), alanine aminotransferase (ALT), C-reactive protein (CRP), erythrocyte sedimentation rate (ESR) and vitamin D. The estimated glomerular filtration rate (eGFR), expressed with the unit mL/min/1.73 m^2^, was calculated by the following formulas:

- Cockcroft–Gault (CG) formula (27,28): eGFR = (140 − age) × weight (kg)/(72 × serum creatinine) × (0.85 if female) [13,14]
- MDRD: eGFR = 175 × (serum creatinine^−1.154^) × (age^−0.203^) × 1.212 (if black) or\and × 0.742 (if female) [15]
- MCQE: eGFR = exp {1.911 + (5.249/serum creatinine) − (2.114/serum creatinine^2^) − 0.00686 × age (−0.205 if female)} [19]
- CKD-EPI (Chronic Kidney Disease Epidemiology): eGFR = 141 × min (Scr/κ,1)^α^ × max (Scr/κ, 1)^−1.209^ × 0.993^Age^ × 1.018 [if female] × 1.159 [if black]; Scr is serum creatinine (mg/dL), κ is 0.7 for females and 0.9 for males, α is −0.329 for females and −0.411 for males, min indicates the minimum of Scr/κ or 1, and max indicates the maximum of Scr/κ or 1 [21].

Participants were classified as stages G1-G5, according to Kidney Disease Improving Global Outcomes (KDIGO) glomerular filtration rate (GFR) categories (1): G1: eGFR ≥ 90 mL/min/m^2^, normal; G2: eGFR 60–90 mL/min/m^2^, mildly decreased; G3a: eGFR 45–59.9 mL/min/m^2^, mildly to moderately decreased; G3b: eGFR 30–44.9 mL/min/m^2^, moderately to severely decreased; G4: eGFR 15–29.9 mL/min/m^2^; severely decreased; G5: eGFR < 15 mL/min/m^2^, kidney failure [32].

The study design of the present investigation was evaluated and approved by the Review Board of the Institute of Medicine and Sports Science. All athletes included in this study were fully informed of the types and nature of evaluation and signed the consent form, according to Italian Law and Institute policy. The data that support the findings of this study are not openly available due to reasons of sensitivity, and are available from the corresponding author upon reasonable request. The data are located in controlled-access data storage at the Institute of Sports Medicine and Science. The work described has been carried out in accordance with The Code of Ethics of the World Medical Association (Declaration of Helsinki).

## 3. Statistical Analysis

Categorical variables were expressed as frequencies and percentages in parentheses and compared using Fisher’s exact test or the Chi-square test, as appropriate. Normality criteria were checked for any continuous variables, which were presented as the mean and standard deviation (SD) and compared using Student’s *t*-test for independent data if normally distributed. All tests were significant if *p* < 0.05. Statistical analysis was performed with STATA Statistics for Windows (SE, version 17).

## 4. Results

We enrolled 490 Olympic athletes, with a mean age of 27 ± 5.3 years and a mean BMI of 22.7 ± 3.2; 292 (59.5) were male, 5.3% (26) were active smokers and 20.8% (102) had a family history of cardiovascular diseases. Athletes practiced different sporting disciplines, divided into skills (182 athletes, 37.1%) and endurance (308 athletes, 62.9%).

Table 1 shows the main differences in clinical and anthropometric parameters and blood test results. Compared to athletes practicing skills disciplines, endurance athletes presented a lower BMI (21.9 ± 3.1 kg/m^2^ vs. 23.9 ± 3.1 kg/m^2^, *p* < 0.0001) and a lower fat mass (13.2 ± 5.3% vs. 20.3 ± 7.8%, *p* < 0.0001), with an accordingly lower prevalence of obesity (0% vs. 6%, *p* < 0.0001). Differential diet composition was noted between the athlete categories, with endurance athletes having a higher daily kcal intake (2807.4 ± 738 vs. 2231.9 ± 482.2, *p* < 0.0001), a higher carbohydrate intake (51.8 ± 5% vs. 49.3 ± 5.2%, *p* = 0.001) and a similar protein intake (*p* = 0.131).

### 4.1. Kidney Function

Endurance athletes showed higher serum creatinine values (0.91 ± 0.14 mg/dL vs. 0.88 ± 0.13 mg/dL, *p* = 0.014) compared to skills athletes. According to the CKD-EPI and MCQE formulas, similar eGFRs were found between the two athlete categories: with CKD-EPI, 121.7 ± 7.9 mL/min × 1.73 m^2^ in skills athletes vs. 121 ± 7.1 mL/min × 1.73 m^2^ in endurance athletes, with *p* = 0.321; with MCQE, 134.5 ± 12.9 mL/min × 1.73 m^2^ in skills athletes vs. 133.8 ± 14.4 mL/min × 1.73 m^2^ in endurance athletes, with *p* = 0.593. Significant differences were found with the CG formula, with endurance athletes showing lower eGFR values: 122.6 ± 30.8 mL/min × 1.73 m^2^ vs. 113.6 ± 27 mL/min × 1.73 m^2^, *p* = 0.008. On the contrary, significant differences were found with the MDRD formula, but endurance athletes showed higher values (129.3 ± 25.8 mL/min × 1.73 m^2^ vs. 122.6 ± 24 mL/min × 1.73 m^2^, *p* = 0.004. Globally, no cases of athletes in the G3b, G4 or G5 categories were observed. According to CKD-EPI, all athletes were in G1, while with MCQE, only one (0.5%) skills athlete and three (1%) endurance athletes were in G2. On the contrary, with the CG formula, a substantial percentage of athletes (of both categories) were in G2 (n = 24, 13.2% for skills, and n = 57, 18.5% for endurance, *p* = 0.125). With the MDRD formula, a total of 29 athletes (5.9%) were in G2 (n = 11, 6% for skills, and n = 18, 5.8% for endurance, *p* = 0.927).

### 4.2. Gender Differences

As shown in Table 2, several significant gender differences were found.

As expected, male athletes presented a different anthropometric profile compared to female athletes, with a higher body weight, higher BMI, higher BSA and lower fat mass % (*p* < 0.0001 in all the above-mentioned parameters and in both sports categories, except for *p* = 0.0006 in the BMI comparison between male and female skills athletes). Significant gender differences in serum creatinine were also noted in both sports categories, with males presenting lower creatinine values (*p* < 0.0001).

With the CKD-EPI and CG formulas, significant gender differences in the eGFR were found. With CKD-EPI, males presented lower eGFR values compared to females: 118.4 ± 6.2 mL/min × 1.73 m^2^ vs. 126.2 ± 7.8. mL/min × 1.73 m^2^, *p* < 0.0001 in skills, and 118.3 ± 6.2 mL/min × 1.73 m^2^ vs. 125.1 ± 6.4 mL/min × 1.73 m^2^, *p* < 0.0001 in endurance. Opposite significant results were found with the CG formula, with females showing a lower eGFR: 107.8 ± 29 mL/min × 1.73 m^2^ vs. 133.2 ± 27.4 mL/min × 1.73 m^2^, *p* < 0.0001 in skills, and 96 ± 26.1 mL/min × 1.73 m^2^ vs. 125 ± 20.7 mL/min × 1.73 m^2^, *p* < 0.0001 in endurance. With the MDRD formula, significant differences were found only in males, with endurance athletes having higher eGFR values (137.5 ± 24.9 mL/min × 1.73 m^2^ vs. 130.3 ± 21.8 mL/min × 1.73 m^2^, *p* = 0.015), whereas similar values were found among female athletes (116.8 ± 21.8 mL/min × 1.73 m^2^ in endurance, vs. 111.7 ± 22.6 mL/min × 1.73 m^2^, *p* = 0.116, in skills). With the MCQE formula, no gender differences were found among skills athletes (*p* = 0.124), while for endurance athletes, males had lower eGFR values (130.5 ± 15.4 mL/min × 1.73 m^2^ vs. 138.8 ± 11.1 mL/min × 1.73 m^2^, *p* < 0.0001). According to the KDIGO categories, with the CG formula, only 4\292 male athletes (1.4%) were in G2, all of whom were endurance athletes (2.1% of endurance athletes vs. 0 skills athletes, *p* = 0.132). In females, 77\198 (38.9%) were in G2, with higher percentages close to statistical significance in endurance athletes (n = 53, 43.3%, vs. n = 24, 31.6%, *p* = 0.095). With the MDRD formula, 7\292 (2.4%) male athletes were in the G2 category (n = 2, 1.9% in skills vs. n = 5, 2.7%, *p* = 0.666), with higher percentages found in female athletes: globally, 22\198 (11.1%) were in G2 (n = 9, 11.8% in skills, vs. n. = 13, 10.7% in endurance, *p* = 0.796).

### 4.3. EGFR Calculated with CG in Female Athletes

As mentioned above, 38.9% of female athletes fell into the KDIGO G2 category when the eGFR was calculated with the CG formula. In Table 3, a comparison of clinical, anthropometric and blood test results is made between female athletes in G2 and G1. Female athletes in G2 were older (28 ± 5 vs. 25.6 ± 5.1 years, *p* = 0.001), with a lower body weight (54.2 ± 6.2 kg vs. 65 ± 12.6 kg, *p* < 0.0001), BMI (19.9 ± 1.8 kg/m^2^ vs. 22.8 ± 3.7 kg/m^2^, *p* < 0.0001), BSA (1.57 ± 0.1 vs. 1.72 ± 0.1, *p* < 0.0001) and fat mass (18.1 ± 4.4% vs. 22.8 ± 3.7%, *p* < 0.0001).

## 5. Discussion

Several creatinine-based estimates of GFR have been proposed to overcome the inadequacy of serum creatinine alone and the problems associated with assessing GFR by urinary clearance of exogenous filtration markers. Such formulas have been extensively evaluated in healthy sedentary populations, whereas data on their use in resting endurance athletes are limited to only a few, relatively small, studies, which include only a subset of sports within this category and, moreover, do not consider all the major formulas at once.

To our knowledge, the present study is the first to evaluate the four main formulas for estimating GFR in a large sample of elite athletes of both genders, representative of almost all disciplines in the endurance category. Endurance athletes were compared with a group of athletes practicing skills disciplines, which are characterized by low cardiovascular engagement and reduced training loads, making them eligible as a control group of healthy individuals. Endurance athletes, as expected based on their high-intensity and long-duration exercise regimens, exhibited a lower BMI, a lower fat mass and, subsequently, a lower prevalence of obesity compared to skills athletes, with no differences in BSA. Previous studies have shown that the serum creatinine concentration is generally higher in athletes than in sedentary people, albeit with differences in creatinine levels among athletes of different sports characterized by varying aerobic or anaerobic components, training loads, length, frequency and competition periods [33,34]. BMI seems to play a relevant role in this context, as it has been observed, initially in the general population [35] and subsequently in athletes of both genders competing in miscellaneous sport categories [34,36,37], that there is a direct relationship between BMI and creatinine values, which could explain the creatinine differences between disciplines. However, homeostatic creatinine values are not merely related to body size and to lean body mass, as the latter has been found to be independent of serum creatinine values [38], but also to other physiological mechanisms, such as the volume of distribution in total body water [39]. This interesting finding is corroborated, in our study, by the evidence of significantly lower creatinine values among males in both sports categories, despite them having a higher body weight, higher BMI and lower fat mass percentage than females. In addition, despite their lower BMI, overall, the endurance athletes showed higher serum creatinine values compared to skills athletes. This result contrasts with two previous studies, although they did not include the full range of endurance disciplines, being limited to a lower number of male athletes practicing exclusively cycling or cross-country skiing [40,41].

Our investigation attests that the application of the four most widely used GFR formulas yields significant variations in the estimated GFR in a large population of endurance athletes of different sports.

As previously demonstrated in sedentary populations [9,12,17], our data add that, even among athletes, CG is particularly influenced by individual anthropometric characteristics: endurance athletes, who have a significantly lower prevalence of obesity than skills athletes, are more likely to incur an underestimation of the GFR compared with the latter. Such underestimation is also confirmed by the comparison within the same gender and the comparison between the two genders, whereby female athletes, characterized by a significantly lower weight and BMI, showed a lower eGFR than male athletes, resulting in more than one-third of the athletes falling into the KDIGO eGFR G2 category based on the CG formula (Table 4).

With regard to the MDRD equation, our results are in agreement with those of a previous study, restricted to a population of male cyclists compared with healthy, sedentary individuals, in which a significantly higher MDRD-estimated GFR was observed in athletes than in controls [42]. In our investigation, we observed significantly higher MDRD-estimated GFR values in endurance athletes, especially among males, compared with those practicing skills disciplines, who represented a less trained population. This observation suggests that the MDRD equation might be influenced more by the intensity and type of physical exercise, whereas CKD-EPI and MCQE were found to be immune to this influence, producing similar eGFR values for the two athlete categories. Furthermore, in agreement with previous evidence in individuals with and without kidney disease [12], we also confirmed, in elite athletes, a tendency for MDRD to underestimate the GFR in females, as revealed by the comparison of male and female athletes.

Hence, our research allow us to provide some certainly useful recommendations for daily practice in the medical sports management of athletes and sportsmen. First, the use of CG in athletes should be discouraged, as it is strongly influenced by anthropometric parameters, such as weight, but also body composition, which are often extreme in some disciplines. In fact, the inclusion of body weight in the formula can lead to systematic overestimation of the GFR in individuals with a higher fat mass, and underestimation in lean individuals with a low body weight, such as female endurance athletes. These limitations stem from the fact that total body weight, rather than fat-free mass, is used in the CG equation, despite the fact that creatinine generation is almost entirely dependent on muscle mass. Second, the use of MDRD should be avoided, both because it was originally validated on individuals with CKD, who are very different metabolically to the athletic population, and because it is likely to be influenced by a heavy training workload.

Consequently, since the CKD-EPI and MCQE formulas do not seem to be influenced by anthropometric factors and the type and intensity of exercise regimens, they should be preferred over the other formulas for use in athletes.

## 6. Limitations

The present study has some limitations. First, the study was designed to be retrospective, meaning that data collection and analysis were based on previously obtained information.

Second, although the athletes were evaluated during a training period, seasonal variability was not considered specifically for each individual athlete. Specifically, training load and intensity could influence biomarkers such as serum creatinine. Nevertheless, it has been demonstrated that serum creatinine concentration is minimally affected by training and competition [34,43,44,45,46].

Third, the athletes included were exclusively Caucasian, which did not allow us to assess the impact of the racial factor present in some of the formulas; however, this study was not designed for that purpose. Lastly, the lack of comparison with the urinary clearance of exogenous filtration markers, such as inulin, iohexol or iothalamate, which represents the gold-standard approach, is a limitation of the study. Such reference methods, however, due to difficulty of use, cost, radiation exposure and ethical issues, are not recommended for use in healthy populations. The use of alternative biomarkers of kidney function, such as cystatin-C, may provide a more accurate and muscle mass-independent estimate of glomerular filtration rate in athletic populations, and further studies are needed to explore this field.

## 7. Conclusions

Our study underscores the complexities of precisely assessing kidney function in elite endurance athletes, who undergo extensive metabolic adaptation and are subjected to intense training or competition regimens.

**Figure 1:**
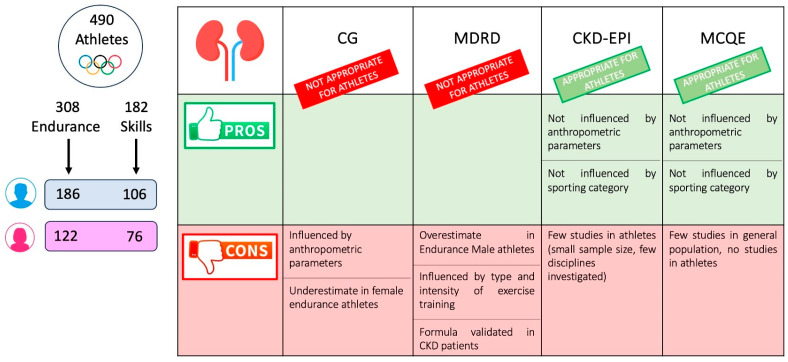
Pros and cons of different creatinine-based formulas in 490 elite athletes practicing endurance (308 athletes) and skills disciplines (182 athletes). CG and MDRD, due to their intrinsic limitations (influenced by anthropometric parameters or exercise training), are not suitable for use in athletes. On the other hand, CKD-EP and MCQE demonstrate greater stability and reliability, and are more appropriate for evaluating kidney function in athletes. Abbreviations: CKD-EPI: Chronic Kidney Disease Epidemiology; CG: Cockcroft–Gault; MCQE: Mayo Clinic Quadratic Equation; MDRD: Modification of Diet in Renal Disease.

Regular monitoring of kidney function is crucial in the medical and sports management of athletes to ensure optimal health outcomes and prevent misdiagnosis of acute or chronic conditions, such as AKI or progressive development of worsening renal function. The comparative evaluation of the four main creatinine-based GFR estimation formulas—CG, MDRD, CKD-EPI and MCQE—revealed significant variations in their efficacy and accuracy within this unique population. Our findings suggest that the CG and MDRD equations are less suitable for athletes, due to their susceptibility to anthropometric and exercise-related variables (Figure 1). In contrast, the CKD-EPI and MCQE formulas demonstrated greater stability and reliability, making them more appropriate for use in athletic populations. Incorporating these recommendations into daily practice can enhance the precision of renal assessment in athletes, ultimately contributing to their overall health and performance.

## Figures and Tables

**Table 1:** Demographic characteristics, anthropometric and clinical parameters and blood test result differences between athletes, according to sporting discipline.

	Skills	Endurance	*p*-Value
n, (%)	182 (37.1)	308 (62.9)	
Male, n (%)	106 (58.2)	186 (60.4)	0.639
Age, mean	27.5 ± 5.5	26.6 ± 4.6	0.086
Weight, kg	72.5.4 ± 11.5	69.1 ± 14.6	0.013
BMI, kg\m^2^	23.9 ± 3.1	21.9 ± 3.1	<0.0001
BSA	1.85 ± 0.21	1.82 ± 0.22	0.171
Fat mass, %	20.3 ± 7.8	13.2 ± 5.3	<0.0001
Smokers, n (%)	25 (13.7)	1 (0.3)	<0.0001
Family history for CVD, n (%)	44 (24.2)	58 (18.8)	0.159
SPB, mmHg	109.0 ± 24.2	107.7 ± 16.9	0.433
DBP, mmHg	68.7 ± 10.9	67.1 ± 10.7	0.152
Obesity, n (%); BMI > 30 kg/m^2^	11 (6)	0 (0)	<0.0001
Kcal	2231.9 ± 482.2	2807.4 ± 738	<0.0001
Protein, %	19.6 ± 4.0	18.8 ± 3.5	0.131
Fat, %	30.2 ± 5.4	29.2 ± 3.3	0.087
Carbohydrate, %	49.3 ± 5.2	51.8 ± 5	0.001
CPK, U/L	178.3 ± 211	260.9 ± 325.3	0.002
AST, U/L	21.3 ± 6.5	29.3 ± 15.5	<0.0001
ALT, U/L	20.7 ± 9.7	24 ± 12.5	0.002
Creatinine, mg/dL	0.88 ± 0.13	0.91 ± 0.14	0.014
CKD-EPI, mL/min × 1.73 m^2^	121.7 ± 7.9	121 ± 7.1	0.321
G2: eGFR 60–89.9 mL/min × 1.73 m^2^	0 (0)	0 (0)	
G1: eGFR ≥ 90 mL/min × 1.73 m^2^	182 (100)	308 (100)	1.000
CG, mL/min × 1.73 m^2^	122.6 ± 30.8	113.6 ± 27	0.0008
G2: eGFR 60–89.9 mL/min × 1.73 m^2^	24 (13.2)	57 (18.5)	0.125
G1: eGFR ≥ 90 mL/min × 1.73 m^2^	158 (86.8)	251 (81.5)	
MCQE, mL/min × 1.73 m^2^	134.5 ± 12.9	133.8 ± 14.4	0.593
G2: eGFR 60–89.9 mL/min × 1.73 m^2^	1 (0.5)	3 (1)	0.614
G1: eGFR ≥ 90 mL/min × 1.73 m^2^	181 (99.5)	305 (99)	
MDRD, mL/min × 1.73 m^2^	122.6 ± 24	129.3 ± 25.8	0.004
G2: eGFR 60–89.9 mL/min × 1.73 m^2^	11 (6)	18 (5.8)	0.927
G1: eGFR ≥ 90 mL/min × 1.73 m^2^	171 (94)	290 (94.2)	

Abbreviations: ALT: alanine aminotransferase; AST: aspartate-transferase; BMI: body mass index; BSA: body surface area; CG: Cockcroft–Gault; CKD-EPI: Chronic Kidney Disease Epidemiology; CPK: creatine phosphokinase; CVD: cardiovascular diseases; DBP: diastolic blood pressure; eGFR: estimated glomerular filtration rate; MCQE: Mayo Clinic Quadratic Equation; MDRD: Modification of Diet in Renal Disease formula.

**Table 2:** Gender differences in anthropometric and clinical parameters and blood test results, according to sporting discipline.

	Male, n = 292			Female, n = 198			Skills	Endurance
	Skills	Endurance	*p*-Value	Skills	Endurance	*p*-Value	Male vs. Female	Male vs. Female
n, (%)	106 (36.3)	186 (63.7)		76 (38.4)	122 (61.6)			
Age, mean	28.2 ± 6.3	26.8 ± 4.5	0.032	26.6 ± 5.8	26.4 ± 4.8	0.871	0.089	0.542
Weight, kg	78.9 ± 12.8	75.7 ± 11.9	0.035	63.5 ± 10.3	59.1 ± 12.4	0.010	<0.0001	<0.0001
BMI, Kg\m^2^	24.6 ± 3.2	22.7 ± 2.6	<0.0001	23 ± 3	20.9 ± 3.4	<0.0001	0.0006	<0.0001
BSA	1.96 ± 0.18	1.94 ± 0.19	0.360	1.69 ± 0.15	1.64 ± 0.13	0.012	<0.0001	<0.0001
Fat mass, %	16.7 ± 7	10 ± 3.3	<0.0001	25 ± 6.1	18 ± 4	<0.0001	<0.0001	<0.0001
Creatinine, mg/dL	0.93 ± 0.1	0.97 ± 0.1	0.048	0.81 ± 0.1	0.84 ± 0.12	0.132	<0.0001	<0.0001
K^+^, mEqu/L	4.47 ± 0.3	4.56 ± 0.3	0.033	4.49 ± 0.4	4.52 ± 0.3	0.549	0.702	0.400
CKD-EPI, mL/min × 1.73 m^2^	118.4 ± 6.2	118.3 ± 6.2	0.891	126.2 ± 7.8	125.1 ± 6.4	0.266	<0.0001	<0.0001
G2: eGFR 60–89.9 mL/min × 1.73 m^2^	0 (0)	0 (0)		0 (0)	0 (0)			
G1: eGFR ≥ 90 mL/min × 1.73 m^2^	106 (100)	186 (100)	1.000	76 (100)	122 (100)	1.000		
CG, mL/min × 1.73 m^2^	133.2 ± 27.4	125 ± 20.7	0.047	107.8 ± 29	96 ± 26.1	0.003	<0.0001	<0.0001
G2: eGFR 60–89.9 mL/min × 1.73 m^2^	0 (0)	4 (2.1)	0.132	24 (31.6)	53 (43.3)	0.095	<0.0001	<0.0001
G1: eGFR ≥ 90 mL/min × 1.73 m^2^	106 (100)	182 (97.9)		52 (68.4)	69 (54.7)			
MCQE, mL/min × 1.73 m^2^	133.3 ± 12.4	130.5 ± 15.4	0.120	136.3 ± 13.3	138.8 ± 11.1	0.145	0.124	<0.0001
G2: eGFR 60–89.9 mL/min × 1.73 m^2^	0 (0)	3 (1.6)	0.187	1 (1.3)	0 (0)	0.204	0.263	0.158
G1: eGFR ≥ 90 mL/min × 1.73 m^2^	106 (100)	183 (98.4)		75 (98.7)	122 (100)			
MDRD, mL/min × 1.73 m^2^	130.3 ± 21.8	137.5 ± 24.9	0.015	111.7 ± 22.6	116.8 ± 21.8	0.116	<0.0001	<0.0001
G2: eGFR 60–89.9 mL/min × 1.73 m^2^	2 (1.9)	5 (2.7)	0.666	9 (11.8)	13 (10.7)	0.796	0.005	0.003
G1: eGFR ≥ 90 mL/min × 1.73 m^2^	104 (98.1)	181 (97.3)		67 (88.2)	109 (89.3)			

Abbreviations: BMI: body mass index; BSA: body surface area; CG: Cockcroft–Gault; CKD-EPI: Chronic Kidney Disease Epidemiology; eGFR: estimated glomerular filtration rate, K^+^: potassium; MCQE: Mayo Clinic Quadratic Equation; MDRD: Modification of Diet in Renal Disease formula.

**Table 3:** Differences in anthropometric and clinical parameters and blood test results in female athletes with mildly reduced eGFR (calculated with Cockcroft–Gault formula) vs. control group, according to sporting discipline practiced.

	Total Female, n = 198			Skills Female, n = 76			Endurance Female, n = 122		
	G2	G1	*p*-Value	G2	G1	*p*-Value	G2	G1	*p*-Value
n, (%)	67 (33.8)	131 (66.2)		24 (31.6)	52 (68.4)		53 (43.4)	69 (56.6)	
Age, mean	28 ± 5	25.6 ± 5.1	0.001	28.3 ± 5.8	25.8 ± 5.7	0.077	27.8 ± 4.6	25.4 ± 4.6	0.005
Weight, kg	54.2 ± 6.2	65 ± 12.6	<0.0001	55.2 ± 6.1	67.4 ± 9.6	<0.0001	53.7 ± 6.1	63.3 ± 14.3	<0.0001
BMI, Kg\m^2^	19.9 ± 1.8	22.8 ± 3.7	<0.0001	20.7 ± 1.5	24 ± 3	<0.0001	19.6 ± 1.8	21.8 ± 4	0.0003
BSA	1.57 ± 0.1	1.72 ± 0.1	<0.0001	1.58 ± 0.1	1.74 ± 0.1	<0.0001	1.56 ± 0.1	1.70 ± 0.1	<0.0001
Fat mass, %	18.1 ± 4.4	22.8 ± 3.7	<0.0001	21.1 ± 4.5	26.7 ± 5.9	0.0001	16.7 ± 3.6	19.1 ± 3.9	0.0009
Smokers, n (%)	4 (6)	3 (2.3)	0.184	4 (16.7)	3 (5.8)	0.126	0 (0)	0 (0)	1.000
Family history for CVD, n (%)	12 (17.9)	25 (19.1)	0.841	6 (25)	11 (21.1)	0.708	6 (11.3)	14 (20.3)	0.184
SPB, mmHg	99.8 ± 9.8	105.1 ± 9.1	0.0005	98.7 ± 7.9	106.2 ± 7.6	0.0007	100.2 ± 10.5	104.3 ± 10.1	0.049
DBP, mmHg	64.5 ± 6	65.9 ± 7.1	0.209	64.2 ± 5.5	67.1 ± 6.4	0.091	64.6 ± 6.2	64.9 ± 7.5	0.857
Kcal	2238.3 ± 386	2234.5 ± 417	0.965	1996.9 ± 338	2030 ± 385	0.807	2381.5 ± 338	2348.1 ± 390	0.743
Protein, %	18.5 ± 2.5	19.3 ± 5.4	0.349	20.6 ± 2.3	19.3 ± 6.3	0.462	17.3 ± 1.6	19.4 ± 4.9	0.037
Fat, %	29.2 ± 3.3	28.4 ± 6	0.453	29.6 ± 4.2	28.5 ± 8.2	0.652	29 ± 2.7	28.3 ± 4.4	0.536
Carbs, %	52 ± 3.9	50.5 ± 6.7	0.206	49.6 ± 4	48.5 ± 6.1	0.549	53.5 ± 3	51.7 ± 6.7	0.213
CPK, U/L	162.3 ± 104.8	172.9 ± 234.2	0.710	151.3 ± 78.5	110.5 ± 62.3	0.018	167.3 ± 114.4	220 ± 296.9	0.227
AST, U/L	25.1 ± 8.9	22.2 ± 7.8	0.015	21.4 ± 5.7	18.1 ± 4.6	0.019	26.8 ± 9.5	25.3 ± 8.3	0.361
ALT, U/L	21.7 ± 9.9	18.2 ± 8.6	0.009	19 ± 8.1	15.7 ± 5.4	0.044	23 ± 10.4	20.1 ± 10	0.130
Creatinine, mg/dL	0.91 ± 0.1	0.78 ± 0.1	<0.0001	0.93 ± 0.12	0.76 ± 0.09	<0.0001	0.90 ± 0.1	0.80 ± 0.1	<0.0001
CG, mL/min × 1.73 m^2^	79.2 ± 7	114.1 ± 27.7	<0.0001	78.8 ± 6.7	121.2 ± 25.4	<0.0001	79.4 ± 7.1	108.8 ± 28.1	<0.0001
Urine gravity, g/mL	1020.3 ± 6.5	1021.4 ± 8.1	0.515	1018 ± 6.2	1022.5 ± 8.4	0.124	1021.3 ± 6.3	1020.7 ± 7.8	0.767

Abbreviations: ALT: alanine aminotransferase; AST: aspartate-transferase; BMI: body mass index; BSA: body surface area; CG: Cockcroft–Gault; CPK: creatine phosphokinase; CVD: cardiovascular diseases; DBP: diastolic blood pressure.

**Table 4:** Comparison of main results and differences in eGFR and KDIGO categories according to different serum creatinine-based formulas. Abbreviations: CG: Cockcroft–Gault; CKD-EPI: Chronic Kidney Disease Epidemiology; KDIGO: Kidney Disease Improving Global Outcomes; MCQE: Mayo Clinic Quadratic Equation; MDRD: Modification of Diet in Renal Disease formula. ↓ reduced; ↑ increased; = equal.

	CG	CKD-EPI	MCQE	MDRD
Skills vs. endurance	↓ endurance	=	=	↑ endurance
KDIGO category, global	16.5% G2	0% G2	0.8% G2	5.9% G2
KDIGO category, males	1.4% G2	0% G2	1% G2	2.4% G2
KDIGO category, females	38.9% G2	0% G2	0.5% G2	11.1% G2
Male vs. female	↑ males	↓ males	↓ male endurance	↑ males
Male skills vs. endurance	↓ endurance	=	=	↑ endurance
Female skills vs. endurance	↓ endurance	=	=	=

## Data Availability

The data that support the findings of this study are not openly available due to reasons of sensitivity, and are available from the corresponding author upon reasonable request. The data are located in controlled-access data storage at the Institute of Sports Medicine and Science.
